# Saliency Detection of Light Field Images by Fusing Focus Degree and GrabCut

**DOI:** 10.3390/s22197411

**Published:** 2022-09-29

**Authors:** Fuzhou Duan, Yanyan Wu, Hongliang Guan, Chenbo Wu

**Affiliations:** 1 Engineering Research Center of Spatial Information Technology, MOE, Capital Normal University, 105 West Third Ring North Road, Haidian District, Beijing 100048, China; 2 Key Lab of 3D Information Acquisition and Application, MOE, Capital Normal University, 105 West Third Ring North Road, Haidian District, Beijing 100048, China; 3 Academy for Multidisciplinary Studies, Capital Normal University, 105 West Third Ring North Road, Haidian District, Beijing 100048, China

**Keywords:** salient object detection, light field, foreground matting, GrabCut

## Abstract

In the light field image saliency detection task, redundant cues are introduced due to computational methods. Inevitably, it leads to the inaccurate boundary segmentation of detection results and the problem of the chain block effect. To tackle this issue, we propose a method for salient object detection (SOD) in light field images that fuses focus and GrabCut. The method improves the light field focus calculation based on the spatial domain by performing secondary blurring processing on the focus image and effectively suppresses the focus information of out-of-focus areas in different focus images. Aiming at the redundancy of focus cues generated by multiple foreground images, we use the optimal single foreground image to generate focus cues. In addition, aiming at the fusion of various cues in the light field in complex scenes, the GrabCut algorithm is combined with the focus cue to guide the generation of color cues, which realizes the automatic saliency target segmentation of the image foreground. Extensive experiments are conducted on the light field dataset to demonstrate that our algorithm can effectively segment the salient target area and background area under the light field image, and the outline of the salient object is clear. Compared with the traditional GrabCut algorithm, the focus degree is used instead of artificial Interactively initialize GrabCut to achieve automatic saliency segmentation.

## 1. Introduction

Light field represents the propagation of light in all directions in three-dimensional space, and it is used to describe the radiative transfer characteristics of light in real space [1]. Since Gershun proposed it in 1936, researchers have never stopped exploring it [2,3,4,5]. The early way of recording the light field was through a single camera continuously changed its position through a mechanical arm. It was difficult to capture an entire light field with time inconsistency. Ren (2005) released the first handheld light field camera with the continuous development of imaging device technology. This light field camera’s feature of taking pictures first and then focusing attracted more and more scholars to join the light field technology research field [6]. Salient object detection (SOD) is an algorithm that allows the fast acquisition of regions of interest in images [7]. This algorithm can accelerate the process of other advanced computer vision processing tasks such as object recognition and image compression. Therefore, SOD based on light field cameras is highly significant in computer vision tasks.

**Figure 1:**
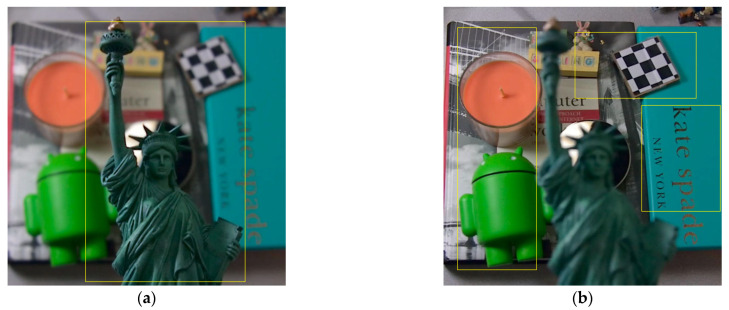
Two of the focus-stacked images: (**a**) Focus on the foreground; (**b**) Focus on the background.

Figure 1 shows that a light field camera can generate refocus images of different focal depth planes through digital refocusing technology. Based on this, a complete traditional RGB image can be obtained by using multifocal fusion technology [8,9]. At the same time, the depth map of the scene can be obtained through multi-view [10] and epipolar plane image (EPI) [11], which allows the use of traditional SOD methods on light field cameras. The focus-stacked images of the light field are used to find images focused on salient objects to generate focus clues. The foreground and background information of the image can be separated using the focus cues to generate a saliency map. The saliency map can provide feature information such as the depth associated with the salient object and constrain the salient areas of the scene [12,13].

GrabCut is a semi-automatic binary classification algorithm [14]. Most scenes only need to provide some seed points to achieve accurate segmentation, and the focus clue of the light field implies the seed point information of GrabCut. Combining the advantages of both, a SOD algorithm based on a light field camera is proposed. We compare the results of 2D, 3D, and light field SOD algorithms and the traditional GrabCut algorithm on existing light field datasets, demonstrating the accuracy of our method in focus calculation, foreground image extraction, and saliency results. The contribution of this work is twofold:

- Aiming at the problem of inaccurate focus area obtained by the focus degree calculation method based on the spatial domain in the existing light field SOD and the problem that the generated focus degree cues have redundant information, we propose a focus degree calculation method based on secondary blurring.
- In this paper, we use the foreground and background information contained in the focus cues of the light field to guide GrabCut to generate foreground color space models and background color space models, respectively. This method enhances the interaction between focus cues and colors and compensates for the drawback that GrabCut requires manual interaction.

The organization of this paper is as follows: Section 2 presents the related work of light-field SOD to summarize existing methods and suggest ideas for improvement. Section 3 introduces the focus calculation method and SOD algorithm proposed in this paper. The experimental setup, evaluation measures, experimental comparisons and experimental results are described in Section 4. Finally, Section 5 discusses the experimental results of this paper.

## 2. Related Works

This section briefly summarizes the SOD algorithms available for light field images based on three image representations: all-in-focus images, depth maps and focus-stacked images.

### 2.1. SOD Based on All-in-Focus Images

A light field camera can generate an all-in-focus image of the scene by taking a photo at one time, while a traditional single-lens reflex (SLR) camera needs to be adjusted to different focal planes multiple times before it can be fused to generate an all-in-focus image of the scene [15,16]. However, the all-focus image is easy to obtain, so the early SOD is mostly based on the 2D SOD algorithm. Itti (1998) first proposed a 2D SOD algorithm based on contrasting features, such as color, brightness and orientation, of RGB images [17]. Later, 2D SOD algorithms were mostly based on the improvement of global contrast or local contrast [18,19,20]. The saliency model that only uses two-dimensional information, such as color, treats the background with the same or similar color as the target object, resulting in the incomplete detection of the target. In order to solve the complex background problem, Yang (2013) and Zhu (2014) used the background before separating the foreground and background in the scene, reducing the background interference [21,22]. Although 2D SOD algorithms are constantly innovating, 2D images only contain light intensity information, so a single clue makes these 2D SOD algorithms require much prior knowledge. At the same time, it is difficult for these algorithms to obtain clean saliency maps in scenes with cluttered backgrounds and similar foreground and background.

### 2.2. SOD Based on Depth Images

The study by Poggio indicated that 2D-based RGB images ignore the human eye’s perception of scene depth [23]. The depth map contains depth information at any point in the scene, making up for the structural information between objects lost in the RGB image [14]. With the development of 3D cameras, such as Kinect, many depth map-based 3D saliency algorithms have emerged [24,25]. Jost believed that the smaller the depth value, the closer to the target, and using the depth information to post-process the 2D saliency results can distinguish the multiple saliency targets of the scene [26]. Ran considered the global depth structure and generated saliency maps based on the differences between the center and surrounding items of the depth map [27]. Peng proposed a multi-level RGBD saliency detection algorithm based on color and depth cues [28]. The RGBD saliency model proposed by Ren utilizes different global priors, such as background priors and depth priors [22]. These 3D saliency detection methods show that the spatial structure information implied in the depth information can distinguish objects at different depth layers in the scene, improving the results of saliency detection to a certain extent. These 3D SOD algorithms are mainly guided by depth, so when the depth map quality is poor, or the depths of the foreground and background are relatively close, 3D SOD cannot be used to generate accurate saliency maps.

### 2.3. SOD Based on Focus Stack Images

A focus-stacked image is a collection of images of different focal planes in a scene generated from light field data by digital refocusing techniques. If you can find an image that is focused near a salient area, it is easier to measure its salience based on the degree of focus. Therefore, Li first proposed a light-field SOD algorithm utilizing the focal feature of focused-stack images, but simply linearly fusing color cues and focality cues could not provide full play to their interaction [29]. Wang (2017) used the gradient value as the focus result based on the spatial domain, and the generated saliency map was close to the result of the method based on the frequency domain, which reduced the calculation time of the focus cue, but the gradient was sensitive to the edge information of the image, which was difficult to detect. The focus information of the background is suppressed [30]. Later, he (2017) proposed saliency maps generated by a probabilistic fusion of different cues using a Bayesian framework [31]. To a certain extent, this makes up for the inaccurate detection in different scenarios caused by the fixed weight value when the previous algorithm adopts the linear weighted fusion method. Zhou (2018) adopted the method of multiple K-Means clustering to fuse focus cues and color cues, and the focus message is required in this method [32]. Piao (2019) guided a cellular automata model based on depth information to generate saliency maps combined with color cues [33]. These light field SOD methods demonstrate the effectiveness of the focus cues provided by light field focus-stacked images to help light field cameras to generate high-quality saliency maps. However, these methods generate focus cues by fusing multiple foregrounds or background images. In the foreground and background with similar depths, the fusion of the focus results of multiple images is prone to clue redundancy, which makes the generated saliency map contours indistinguishable. The chain block effect is pronounced. In addition, the frequency domain-based focus calculation method in the existing light field saliency model has a high time complexity, while the spatial domain-based focus calculation method is calculated by the gradient. For the out-of-focus region of the focal image, the detection result produces a certain error if the edge information is rich.

## 3. Methodology

This section introduces our fused focus degree with GrabCut for light field image SOD. The input images to our algorithm were focus-stacked images (depth numbers d = 1, 2, ..., L) and all-in-focus images. The focus-stacked image contains depth information, and the foreground and background images located at different depth layers are extracted according to the background connectivity detection. Therefore, corresponding to the foreground and background area in the all-focus image, respectively, the color Gaussian mixture model of the foreground and background of GrabCut was constructed. Next, the saliency map was iteratively calculated based on the maximum flow minimum cut algorithm of graph cuts.

### 3.1. Extracting Focusness Information from Focus Stack

**Figure 2:**
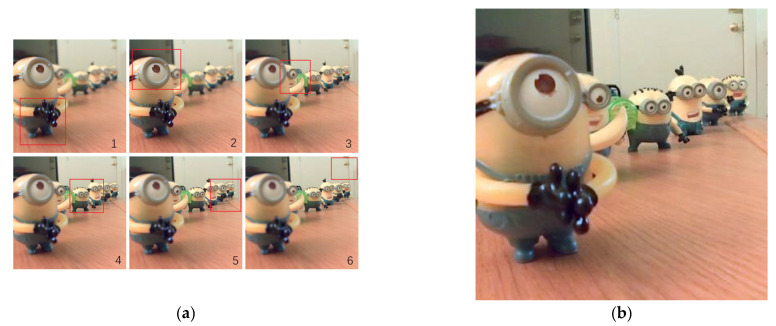
(**a**) Focus-stacked images. (**b**) All-in-focus images.

As seen in Figure 2, in the sequence of focus-stacked images, the depth position of the clear region moves from front to back as the sequence number of the focal slice increases. For optical field saliency detection, the focal degree of focus stack images is mostly calculated from the frequency domain [31,32,33]. Although the frequency domain-based focal degree calculation method can obtain more accurate high-frequency components of the images, it has a relatively high time complexity. In a recent light field-based SOD work [30], the focus was measured in the spatial domain by using a gradient operator, which is much faster to process. However, the gradient is sensitive to the grayscale changes between adjacent pixels in the image, and even in the defocused area, certain gradient information is generated. In order to suppress the gradient information in the defocused regions, we propose to perform secondary (multiple) blurring on the original image. It makes the difference of gradient change before and after the focused region of the original image greater and the difference of gradient change in the bokeh region smaller, which makes it easier to enhance and distinguish the results of the focused region and the defocused region in comparison.

Before using the gradient operator to measure the sharpness of the focus-stacked image, we needed to degrade it. Let $\;\left(i,\;j\right)\;$ denote the pixel in the image from the focus stack. i=1,2, …,w;j=1,2, …,h;w, h are the width and height of input image, respectively.$ReBlur\left(i,j\right)$ is the RGB value of the pixel $\left(i,\;j\right)$ calculated by the mean filter:

(1) $$Reblur\left(i,j\right)=Image\cdot w=\overset{1}{\underset{m=-1}{\sum }}\overset{1}{\underset{n=-1}{\sum }}g\left(i+m,j+n\right)$$

where w is a 3 × 3 convolution kernel defined by:

(2) $$w=\left[\begin{array}{ccc} 1/9 & 1/9 & 1/9\\ 1/9 & 1/9 & 1/9\\ 1/9 & 1/9 & 1/9 \end{array}\right]$$

Then,$\;Gray\left(i,j\right)$ is the grayscale value of the pixel $\left(i,j\right)$ calculated by weighting the RGB value of $\left(i,j\right)$:

(3) $$Gray\left(i,j\right)=R\left(i,j\right)\times 0.299+G\left(i,j\right)\times 0.587+B\left(i,j\right)\times 0.114$$

Next, the gradient value $G\left(i,j\right)$ of any pixel $\left(i,j\right)$ of the focus stack image is calculated as:

(4) $$\left\{\begin{array}{c} G_{x}\left(i,j\right)=Gray\left(i+1,j\right)-Gray\left(i,j\right)\\ G_{y}\left(i,j\right)=Gray\left(i,j+1\right)-Gray\left(i,j\right)\\ G\left(i,j\right)=\left|\begin{array}{c} G_{x}\left(i,j\right) \end{array}\right|+\left|\begin{array}{c} G_{y}\left(i,j\right) \end{array}\right| \end{array}\right.$$

The sharpness matrix of each slice in the focus-stacked image can be defined as:

(5) $$F\left(i,j\right)=G_{original}\left(i,j\right)-G_{reblur}\left(i,j\right)$$

where $G_{original}\left(i,j\right)$ and $G_{reblur}\left(i,j\right)$ are denoted as the original image slice and the degraded image slice in the focus-stacked image, respectively. Then, we contrasted and enhanced the obtained $F\left(i,j\right)$ to suppress the sharpness value of the defocused area.

### 3.2. Filtering Foreground/Background Images with Object Priors

Now, we filtered each image in the focus-stacked image to find the focal images that focus on the foreground and background regions’ focus stack. In recent work based on light field SOD, the position of the focal point is measured by the image’s center point as the filter center to pick out the focal image close to the center point of the image. However, salient objects are not always at the center point of the image [17,18,19]. In this paper, we used background connectivity detection [22] to measure the background region of the all-in-focus image, and we also obtained the object region through the inversion operation. We took the center point of the object region as our Gaussian filtered object center $\left(\mu_{x},\mu_{y}\right)$. $Gauss\left(x,\mu_{x}\right)$ represents the result of the sharpness matrix filtered along the coordinate axis *x*:

(6) $$Gauss\left(x,\mu_{x}\right)=\exp\left(-\frac{{\left(x-\mu_{x}\right)}^{2}}{2\sigma^{2}}\right)$$

where $\mu_{x}$ is the center point of the one-dimensional Gaussian filter on the coordinate axis *x*, and σ controls the band width of the filter. Next, we decomposed the sharpness into two one-dimensional sharpness values along the horizontal and vertical directions:

(7) $$\left\{\begin{array}{c} D_{x}=\frac{\sum_{j=1}^{h}F\left(i,j\right)}{\sum_{i}\sum_{j}F\left(i,j\right)}\\ D_{y}=\frac{\sum_{i=1}^{w}F\left(i,j\right)}{\sum_{i}\sum_{j}F\left(i,j\right)} \end{array}\right.$$

Therefore, we computed the foreground measurement:

(8) $$OS\left(I^{k}\right)=\exp\left(\frac{\lambda \cdot \left(-k\right)}{K}\right)\cdot \left[\overset{w}{\underset{x=1}{\sum }}D_{x}^{k}\cdot G\left(x,\mu_{x}\right)+\overset{h}{\underset{y=1}{\sum }}D_{y}^{k}\cdot G\left(y,\mu_{y}\right)\right]$$

where *k* represents the serial number of the focus image and *K* is the total number of focus stack images. The focus-stacked images are sorted by depth from small to large, so the larger the value of −*k*/*K*, the smaller the depth value of the focus image. λ represents the weighting factor of depth. The image with the highest foreground measurement value was selected as the foreground layer, and the sharpness matrix of the foreground layer was denoted as $G_{F}$. The image with the smallest foreground measurement was selected as the background layer, and the sharpness matrix of the background layer was denoted as $G_{B}$. However, when the depth of the scene is close, the focus area of the background layer may still contain foreground information; therefore, if $OS_{min}\geq 0.7\cdot OS_{max}$, the sharpness matrix $G_{B}$ of the background layer is obtained by inverting the sharpness matrix $G_{F}$ of the foreground layer.

### 3.3. Detecting the Foreground/Background Regions Coarsely

We used the selected sharpness measures of the foreground and background layers to distinguish the foreground and background regions in the all-in-focus image, respectively. The focus degree of this paper is not obvious in the area where the adjacent pixels of the focus area does not change much. Through the simple linear iterative cluster (SLIC) superpixel segmentation algorithm [34], the all-focus image is divided into *N* superpixels *r_i_*, *i* = 1, 2, …, *N*. We considered the focus degree of each pixel in the superpixel region to be the same. Therefore, we took the average value of the focus of all pixels in the region as its focus degree, and the focus degree for a superpixel region r can be expressed as:

(9) $$F\left(r\right)=\underset{\left(x,y\right)\in r}{\sum }\frac{F\left(x,y\right)}{A_{r}}$$

where $A_{r}$ represents the number of pixels in the superpixel. Here, we utilized an object Gaussian model to analyze each pixel of the foreground layer. Therefore, foreground cues are calculated as:

(10) $$FC\left(r\right)=F_{F}\left(r\right)\cdot \left(exp^{-\frac{{\left(r-r_{o}\right)}^{2}}{2\sigma_{r}^{2}}}\right)$$

where $r_{o}$ represents the center of the superpixel where the center point $\left(\mu_{x},\mu_{y}\right)$ of the object region is located. Similarly, we analyzed the background layer according to the object biased Gaussian model. Therefore, background cues are calculated as:

(11) $$BC\left(r\right)=F_{B}\left(r\right)\cdot \left(1-exp^{-\frac{{\left(r-r_{o}\right)}^{2}}{2\sigma_{r}^{2}}}\right)$$

where $F_{F}\left(r\right)$ and $F_{B}\left(r\right)$ denote the focus degree of the foreground and background superpixel regions, respectively. Thereafter, we threshold the foreground cues and background cues, respectively, to obtain the foreground and background regions of the all-focus image, whose pixel values are binary and can be regarded as rough foreground saliency map and background saliency map.

### 3.4. Fusioning Focus Cues and GrabCut to Generate Saliency Map

GrabCut is a binary image segmentation algorithm. However, GrabCut requires human interaction to provide foreground or background seed point information to initialize the Gaussian mixture model [14]. In Section 3.3, we obtained the foreground and background regions of the image through focus cues, which provides a new way for the GrabCut algorithm to provide initialization information automatically. Therefore, accurate foreground and background seed point information can make the saliency segmentation result more accurate. We performed morphological operations on images of FC and BC to remove isolated points while optimizing edge information using a conditional random field model.

In GrabCut, the foreground and background are modeled using two fully covariance Gaussian mixture models (GMM) with K components, one for the foreground and one for the background. Then, there are additional vectors $k=\left\{k_{1},\ldots ,k_{n},\ldots ,k_{N}\right\},$ where $k_{N}\in \left\{1,\ldots ,K\right\}$. Assign a unique GMM component to each pixel, according to $a_{n}=0\;or\;1$, determine whether it is the GMM component of the background model or the GMM component of the foreground model. Therefore, the Gibbs energy of the whole image can be expressed as:

(12) $$E\left(\underline{\alpha },k,\underline{\theta },z\right)=U\left(\underline{\alpha },k,\underline{\theta },z\right)+V\left(\underline{\alpha },z\right)$$

where the region term $U\left(\underline{\alpha },k,\underline{\theta },z\right)$ is related to the color Gaussian mixture model and is defined as follows:

(13) $$U\left(\underline{\alpha },k,\underline{\theta },z\right)=-log\pi \left(\alpha_{n},k_{n}\right)+\frac{1}{2}logdet\sum \left(\alpha_{n},k_{n}\right)+\frac{1}{2}{\left[z_{n}-\mu \left(\alpha_{n},k_{n}\right)\right]}^{T}\sum {\left(\alpha_{n},k_{n}\right)}^{-1}\left[z_{n}-\mu \left(\alpha_{n},k_{n}\right)\right]$$

where the initial value of $\alpha_{n}$ is determined by FC and BC that belongs to the GMM of the foreground or background. π is the weight of the Gaussian component, μ is the mean vector of the Gaussian component, and ∑ is the covariance of the Gaussian components. According to the RGB value of the pixel and the parameters of the three Gaussian components, the probability of its foreground or background can be obtained. The boundary term $V\left(\underline{\alpha },z\right)$ is calculated by the Euclidean distance of RGB to measure the similarity of two neighboring pixels:

(14) $$V\left(\underline{\alpha },z\right)=\gamma \underset{\left(m,n\in \right)}{\sum }\left[\alpha_{m}\neq \alpha_{n}\right]exp-\beta \|z_{m}-z_{n}\|{}^{2}$$

where γ is the constant 50 and β is the contrast factor. The total energy $E\left(\underline{\alpha },k,\underline{\theta },z\right)$ is segmented by the max-flow/min-cut algorithm, and the foreground and background region item data of the next iteration are obtained, until the $E\left(\underline{\alpha },k,\underline{\theta },z\right)$ iteration converges to obtain the saliency segmentation result.

## 4. Experiments

In this section, we conduct experiments to evaluate the performance of our algorithm. First, the experimental setup was outlined, including datasets, evaluation metrics, and parameter settings. Next, a quantitative evaluation of our light-field SOD method was compared with the 2D SOD method [22,34,35], the 3D SOD method [36], and the light field SOD method [15]. Finally, the experimental comparison with the traditional GrabCut algorithm was carried out to prove the effectiveness of the focus results in this paper for the interaction-free GrabCut algorithm.

### 4.1. Experimental Setup

#### 4.1.1. Datasets

We conducted SOD experiments on the publicly available light field dataset provided by Li et al. [29]. The dataset was captured by a Lytro light field camera and includes 60 images of indoor scenes and 40 images of outdoor scenes. Each scene contains fully focused RGB images, a focus-stacked image focused at different depths, and a depth image to facilitate testing different saliency models. One part of the dataset has complex background colors and similar foreground colors, and the other contains multiple salient objects.

#### 4.1.2. Evaluation Metrics

In order to quantitatively illustrate the performance of the algorithm, we used three popular SOD quantitative indicators: PR curve, F-Measure, and Mean Absolute Error (MAE) [37], which are introduced in detail next. In the PR curve, P represents the precision rate (*Precision*) and R represents the recall rate (*Recall*). *Precision* and *Recall* are calculated as:

(15) $$\left\{\begin{array}{c} Precision=\frac{sum\left(S,GT\right)}{sum\left(S\right)}\\ Recall=\frac{sum\left(S,GT\right)}{sum\left(GT\right)} \end{array}\right.$$

where sum represents the sum of pixels with a value of 1. S means saliency map GT means ground truth. If the PR curve is closer to the (1,1) position, the higher the precision rate under the same recall rate, the better the algorithm performance.

F-Measure is a weighted harmonic evaluation of precision rate *P* and recall rate *R*. F-Measure can be used to evaluate the performance of the algorithm by combining the two indicators, which is an overall measurement method. The formula of F-Measure is calculated as:

(16) $$F_{\beta }=\frac{\left(1+\beta^{2}\right)P\times R}{\beta^{2}\cdot P+R}$$

According to the description in the literature [37], by setting the weight $\beta^{2}$ to avoid the problem of excessive recall caused by expanding the SOD area, this paper set $\beta^{2}$ to 0.3. The larger the F-Measure, the more stable the algorithm performance.

*MAE* stands for mean absolute error. The smaller the value of *MAE*, the smaller the error with the true value, and the more stable the algorithm is. It is defined as the pixel-averaged absolute difference between the saliency map S and the ground-truth map GT:

(17) $$MAE=\frac{1}{w\times h}\overset{w}{\underset{x=1}{\sum }}\overset{h}{\underset{y=1}{\sum }}\left|S\left(x,y\right)-GT\left(x,y\right)\right|$$

### 4.2. Comparison with Different Methods

For the SOD of light field images, we compared the SOD algorithms of different input data, including three 2D SOD algorithms based on all-focus images, Geodesic Saliency (GS [36]), Manifold Ranking (MR [21]), Saliency Optimization (wCtr [22]); a 3D SOD algorithm based on depth maps and all-focus images, Local Background Enclosure (LBE [38]); and a light-field SOD algorithm based on focus stacks and all-focus images, Light Field Saliency (LFS [29]). To ensure fairness, all saliency results from competing methods were produced by running the source codes or pre-computed by the authors.

#### 4.2.1. Quantitative Evaluation

**Figure 3:**
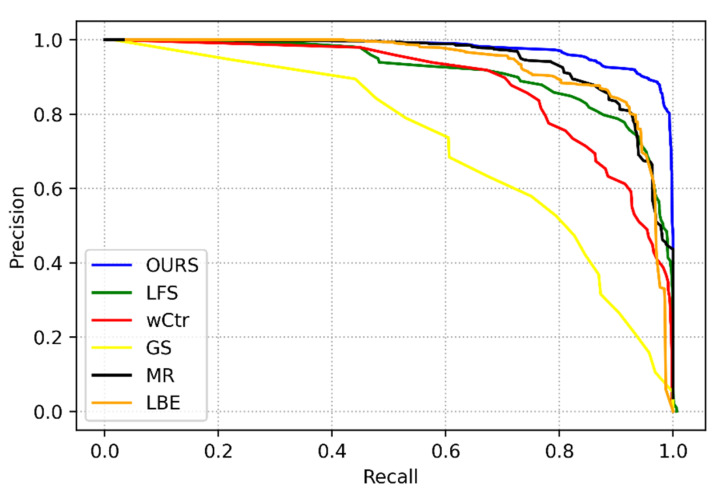
Precision–recall curves of our proposed method and other salient object detections in the light field datasets.

**Figure 4:**
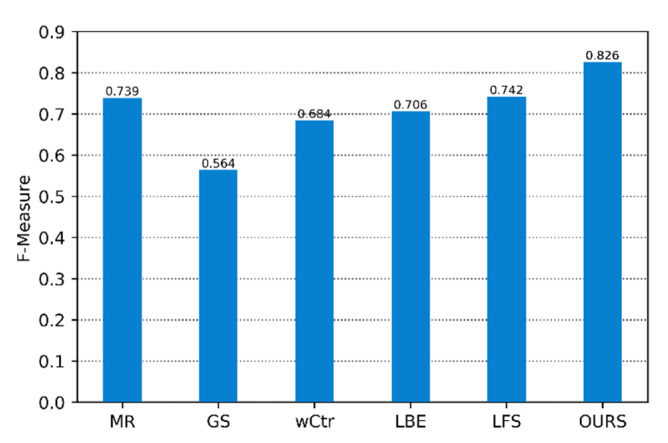
F-Measure of our proposed method and other salient object detections in the light field datasets.

**Figure 5:**
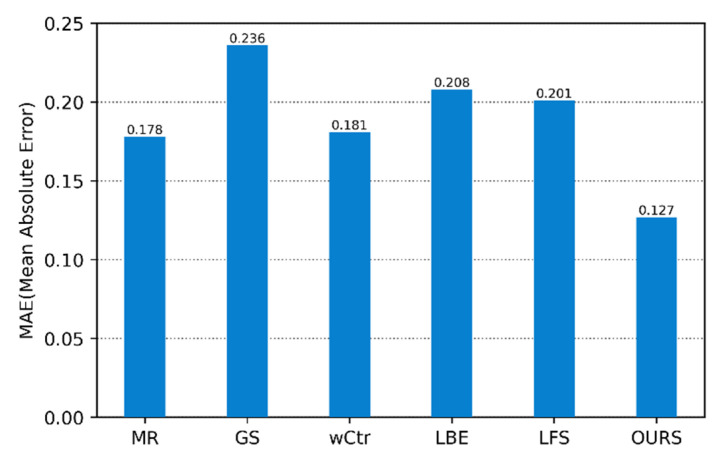
MAE of our proposed method and other salient object detections in the light field datasets.

Figure 3 shows the quantitative evaluation of the method in this paper and other SOD algorithms on the PR curve. Figure 4 and Figure 5 are the comparison of the corresponding F-Measure and MAE values, respectively. According to these three images, it is clear that the algorithm’s performance in this paper is higher than that of other SOD methods on the three evaluation indicators, which shows the superiority of the SOD based on the focus of the light field in this paper. Compared with the SOD algorithm, the fusion method of focus and color cues can better exert their interaction and obtain more accurate saliency results.

#### 4.2.2. Qualitative Evaluation

**Figure 6:**
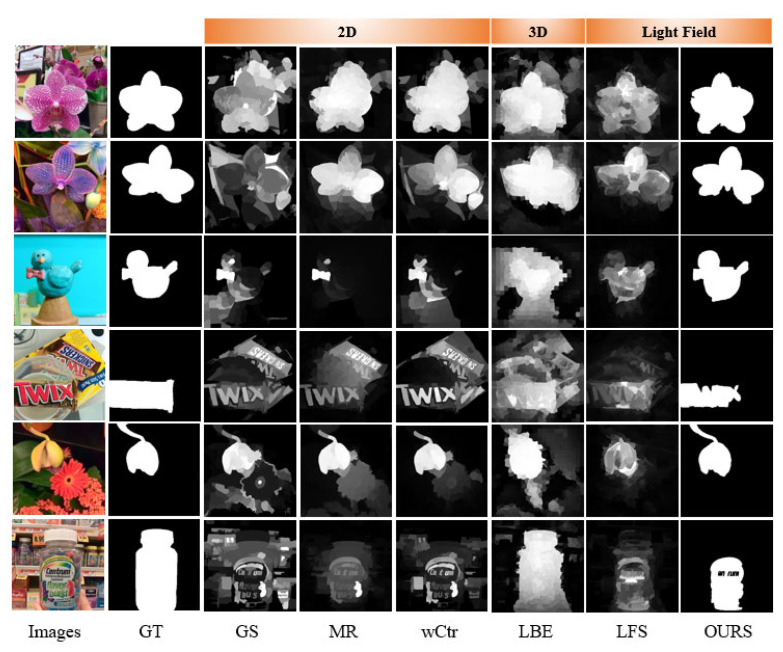
The saliency map obtained by different salient object detection methods.

Figure 6 shows some of the SOD results on the dataset. Most of these are complex scenes with similar foreground and background and cluttered backgrounds. Comparing the detection results with the true value, we can see the algorithm performance’s pros and cons. The 2D SOD algorithm is more susceptible to complex background interference, salient objects cannot be well separated from the background, and the detection results are better when the scene is relatively simple. The 3D SOD algorithms cannot effectively detect salient objects when their depths are close to the background. The light field SOD algorithm achieves a certain separation between the salient objects and the background in the scene according to the focus cue. Compared with other saliency algorithms, the saliency map obtained by this algorithm has a clear outline. Although the algorithm in this paper only recognizes the label on the transparent object (see the sixth row in Figure 6), there are some misjudgments. However, for most salient regions, regardless of whether the foreground and background are similar or whether the scene is complex, this paper’s algorithm can highlight the salient area better. At the same time, with the advantage of GrabCut binary classification, the algorithm in this paper is a complete binary image compared with the saliency map generated by other algorithms.

### 4.3. Validity Analysis of Focus Degrees

In this part, we analyzed the focus degree results generated by the direct gradient method and the gradient method in this paper, including the degree of suppression of the defocused area and the results generated by the fusion GrabCut. At the same time, we also compared with the results generated using the traditional GrabCut algorithm.

#### 4.3.1. Analysis of the Defocused Area Suppression Degree of the Focus Image

The quality of the focus results affects the accuracy of the focus cues. Therefore, to verify the feasibility of using the secondary blurring method to calculate the gradient change proposed in this paper, we compared the approach of this paper with that of Wang [28]. We calculated the average number of pixels in the defocused area in the foreground of some different scenes with $F\left(i,j\right)\;$> 0. As shown in Table 1, we can see that the average number of pixels in the defocused area obtained by the secondary blurring method in this paper is reduced by 30% compared with the average number of pixels in the defocused area calculated by the direct gradient method. The experimental comparison verifies that the method based on secondary blurring to calculate the focus degree in this paper has a good suppression effect on the number of pixels in the defocused area compared with the method of gradient directly as the focus degree. This method has certain advantages in calculating the focus degree clues.

#### 4.3.2. Accuracy Analysis of Extracted Foreground Images

A foreground image is a target region of interest extracted from an image, and the target region is generally the focused region of the image, while the defocused region can be regarded as the background image. If accurate foreground and background images can be obtained, the accuracy can be significantly improved in the subsequent SOD tasks.

Suppose I^k^ is a focal image in the focus stack image. Assume I^k^ is a focal image in the focus-stacked image. We computed the foreground score values for the focus-stacked image sets I^1^ to I^11^ according to Equation (8) using the focus degree algorithm in the existing light field saliency model and our focus degree algorithm, respectively, and the results are shown in Table 2.

From Table 2, we can see that the time consumption of this method and that of the gradient-based focus calculation method are close, but both are faster than the frequency-domain-based focus calculation method. The frequency domain-based focus calculation has a higher time complexity because the image is converted to the frequency domain for DCT transformation. Finally, in the foreground scoring value, the frequency domain-based method does not vary significantly in the scoring value of each focus image. This is because the frequency domain is more sensitive to high frequencies, and in complex scenes, there may be high-frequency responses even in the scattered focus region, so the focus images of different depths may produce high-frequency responses in the target region. However, the gradient-based and our method significantly differ in the focus images at different depths.

#### 4.3.3. SOD Results Using GrabCut with Different Focus Methods

**Figure 7:**
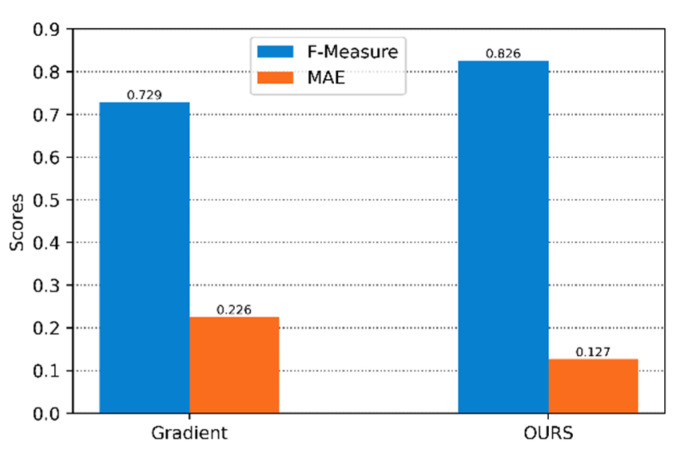
F-Measure and MAE obtained by GrabCut for two focus calculation methods.

We calculated its two evaluation indicators, F-Measure and MAE, on the LFSD dataset, as shown in Figure 7. According to the F-Measure and MAE values, it can be seen that the results generated by the GrabCut calculation method based on the focus degree in this paper are higher than the calculation method based on the gradient as the focus degree in both evaluation indicators.

**Figure 8:**
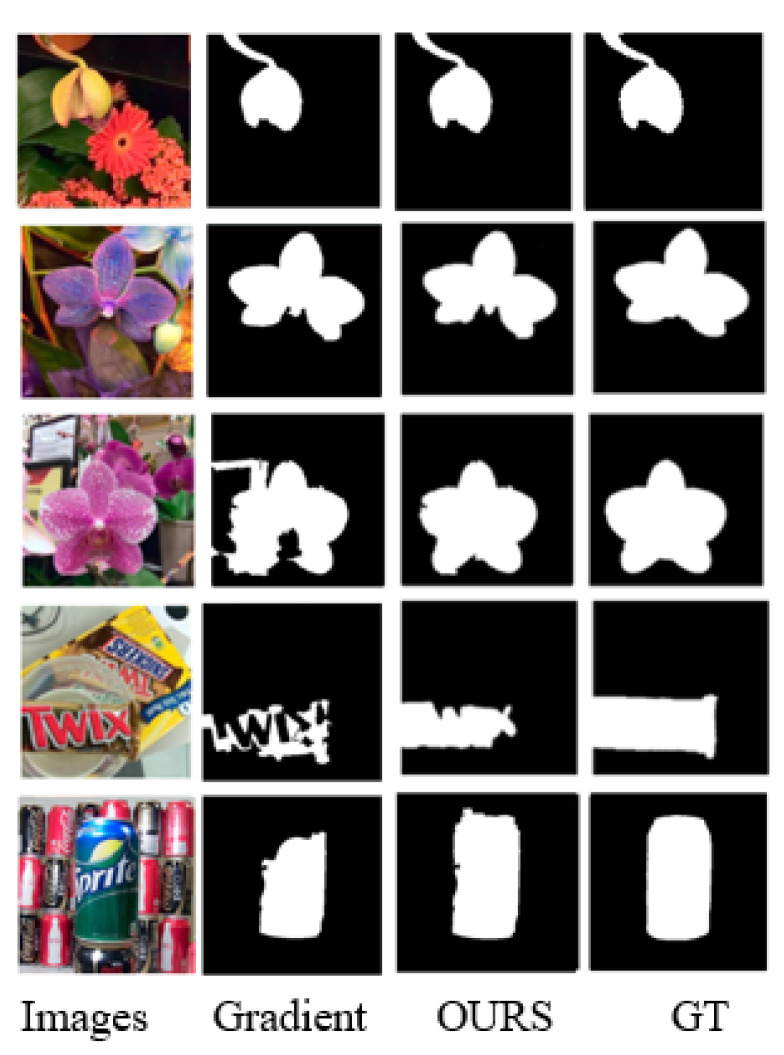
The saliency map obtained using GrabCut for two focus calculation methods.

Figure 8 shows the partial results of the two focusing degree calculation methods using GrabCut to calculate the saliency. It can be seen from the first and second rows that, in a simple scene, the saliency results obtained by the two focusing degrees can accurately highlight objects. In the complex scene where the third to fifth rows are located, the difference in the focus calculation method generates different focus cues, making the seed points of the color space model in the GrabCut algorithm different, resulting in the unclear segmentation of significant objects. To sum up, the focus calculation method proposed in this paper suppresses the focus information of the defocused area and reduces the interference of the background area to the salient objects.

#### 4.3.4. Comparison with the Traditional GrabCut Algorithm

**Figure 9:**
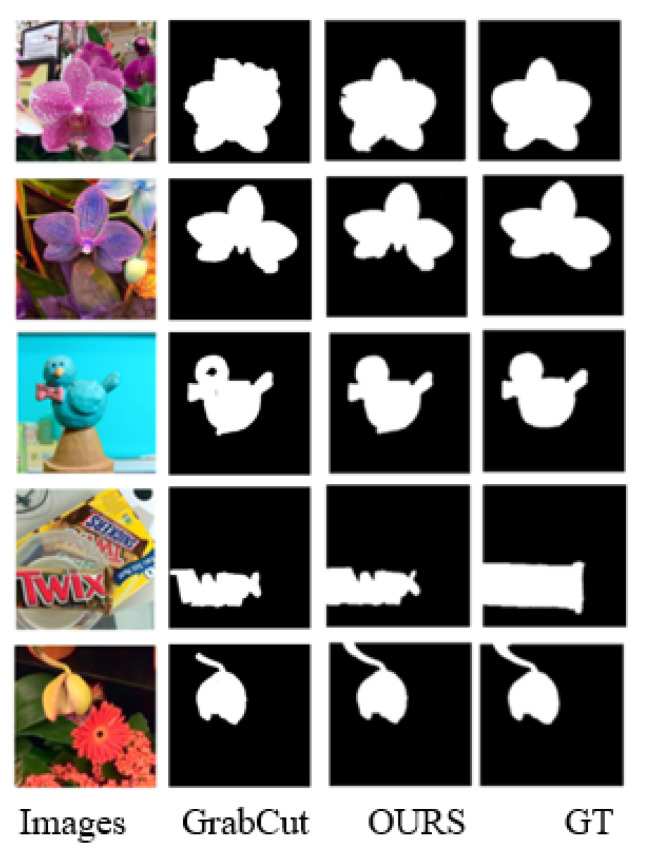
The saliency map obtained by our method and traditional GrabCut.

Using human interaction, we generated saliency maps for GrabCut annotating foreground and background regions. Figure 9 shows the comparison between the results of our method and the traditional GrabCut method. Although the boundary information of foreground and background is provided by manual interaction, it can be seen from the results in the third row that there are still some missed detections in the interior of the salient objects. At the same time, in the fifth row, from the root of the flower, it can be seen that, if the seed point is not fully labeled at the edge, the missed detection still occurs, and the focus clue provided in this paper is the focus result of the light field focus-stacked image at the foreground and background depths.

**Figure 10:**
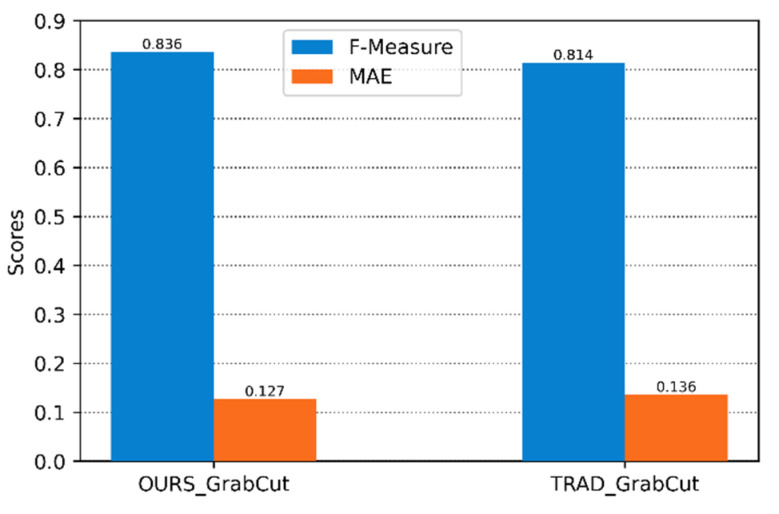
F-Measure and MAE obtained by our method and traditional GrabCut.

Figure 10 compares the F-Measure and MAE values generated by the GrabCut algorithm with and without manual interaction. The results of this paper are slightly higher than those of the manual interaction method in the evaluation of the two indicators. It can be seen that the focus clue provided in this paper is significant for GrabCut interaction-free effectiveness.

## 5. Discussion

In this paper, we proposed a light field salient object detection algorithm that integrates focus and GrabCut, suppressing redundant information of focus cues and making the generated saliency map clear. In addition, in order to adapt to light field saliency detection in different complex scenes and maximize the complementarity of focus cues and color cues, the algorithm proposed in this paper combines the characteristics of the GrabCut algorithm to use focus cues to guide color cues to establish an initialized color space model. Interaction-free saliency detection for the foreground of light field images was achieved. The experimental results show that the proposed algorithm can achieve better saliency detection results in different light field scenarios compared with other algorithms by testing in the public light field dataset. Finally, through experimental comparison with the traditional manual interactive GrabCut algorithm, the seed point information provided by the focus of the light field is not much different from the saliency results generated by the manually marked seed point information, achieving the advantages of the focus of the light field and GrabCut.

However, our method still has certain shortcomings. The effect of the algorithm in this paper is more dependent on the accuracy of the focus cues. Although reliable seed point information can be provided in most scenes, when the edge information of the focus area of the transparent object or the focus image is not rich, the focus result information of this paper is not enough. Some, although few, scenes will be missed. In Figure 6, the transparent objects make the model misjudge the background information and only detect the label area of the bottle. We believe that subsequent references to depth maps can help focus cues to determine thresholds.

## Figures and Tables

**Table 1:** The number of pixels in some foreground images of different focus calculation methods.

Focus Method	Focused Area Pixels	Defocused Area Pixels
gradient	8439	2492
ours	5697	1583

**Table 2:** The calculation result of the foreground score value of the focus image.

	OS(I^1^)	OS(I^2^)	OS(I^3^)	OS(I^4^)	OS(I^5^)	OS(I^6^)	OS(I^7^)	OS(I^8^)	OS(I^9^)	OS(I^10^)	OS(I^11^)	Time
frequency domain-based method	0.826	0.876	0.855	0.804	0.781	0.758	0.736	0.715	0.695	0.675	0.656	8.758
gradient-based method	0.796	0.849	0.845	0.680	0.646	0.578	0.510	0.464	0.431	0.418	0.425	6.303
ours	0.961	0.975	0.977	0.850	0.772	0.563	0.436	0.362	0.333	0.324	0.325	6.430

## Data Availability

The data presented in this study are available on request from the corresponding author. The data are not publicly available due to privacy reason.
